# Natalizumab promotes activation and pro-inflammatory differentiation of peripheral B cells in multiple sclerosis patients

**DOI:** 10.1186/s12974-019-1593-2

**Published:** 2019-11-16

**Authors:** Jan W. Traub, Hannah L. Pellkofer, Katja Grondey, Ira Seeger, Christoph Rowold, Wolfgang Brück, Leila Husseini, Silke Häusser-Kinzel, Martin S. Weber

**Affiliations:** 1 Institute of Neuropathology, University Medical Center, Robert-Koch-Straße 40, 37099 Göttingen, Germany; 2 Department of Neurology, University Medical Center, Robert-Koch-Straße 40, 37099 Göttingen, Germany; 3 Institute of Clinical Neuroimmunology, Ludwig Maximilian University, Großhaderner Straße 9, 82152 Munich, Germany

**Keywords:** Multiple sclerosis, Natalizumab, B cells, Dimethyl fumarate, Glatiramer acetate, Fingolimod

## Abstract

**Background:**

In the past, multiple sclerosis (MS) medications have been primarily designed to modulate T cell properties. Based on the emerging concept that B cells are equally important for the propagation of MS, we compared the effect of four commonly used, primarily T cell-targeting MS medications on B cells.

**Methods:**

Using flow cytometry, we analyzed peripheral blood mononuclear cells (PBMC) of untreated (*n* = 19) and dimethyl fumarate (DMF; *n* = 21)-, fingolimod (FTY; *n* = 17)-, glatiramer acetate (GA; *n* = 18)-, and natalizumab (NAT; *n* = 20)-treated MS patients, focusing on B cell maturation, differentiation, and cytokine production.

**Results:**

While GA exerted minor effects on the investigated B cell properties, DMF and FTY robustly inhibited pro-inflammatory B cell function. In contrast, NAT treatment enhanced B cell differentiation, activation, and pro-inflammatory cytokine production when compared to both intraindividual samples collected before NAT treatment initiation as well as untreated MS controls. Our mechanistic in vitro studies confirm this observation.

**Conclusion:**

Our data indicate that common MS medications have differential, in part opposing effects on B cells. The observed activation of peripheral B cells upon NAT treatment may be instructive to interpret its unfavorable effect in certain B cell-mediated inflammatory conditions and to elucidate the immunological basis of MS relapses after NAT withdrawal.

**Trial registration:**

Protocols were approved by the ethical review committee of the University Medical Center Göttingen (#3/4/14).

## Background

The enormous success of B cell-depleting anti-CD20 antibodies in treatment of multiple sclerosis (MS) [1] corroborates that B cells play an important role in its pathogenesis. Antigen-activated, differentiated B cells most likely act as potent antigen-presenting cells for the activation of T cells and as providers of pro-inflammatory cytokines [2]. Hereby, B cells are considered to be crucial for the development of new inflammatory central nervous system (CNS) lesions and acute MS relapses.

Based on the earlier assumption that the pathophysiology of MS is mainly mediated by T cells, the majority of established MS medications have been designed and trialed to target pro-inflammatory T cell properties. As a consequence, relatively little is known on how these MS drugs may influence B cells, and if so, how this may contribute to their therapeutic efficacy. Therefore, we investigated in a parallel approach how the four commonly prescribed medications dimethyl fumarate (DMF), fingolimod (FTY), glatiramer acetate (GA), and natalizumab (NAT) affect peripheral B cells with a focus on B cell activation, differentiation, and cytokine production, a procedure allowing us to directly compare treatment effects without inter-study discrepancies.

## Methods

### Study subjects and sample preparation

We collected peripheral blood from relapsing-remitting MS patients at the Clinical MS Center of the University Medical Center Göttingen (#3/4/14) between 2015 and 2018. Patients had received DMF, FTY, GA, and NAT according to the current European guidelines [3] for the respective drug for at least 6 months or had not received any permanent medication or steroids for at least 6 months (untreated controls). Seven patients receiving NAT were in addition analyzed longitudinally. Demographic and disease-related information including age, gender, disease activity, disease onset, treatment duration, and previous treatments of the patient cohorts are summarized in Table 1. Human peripheral blood mononuclear cells (PBMC) were isolated by Ficoll density gradient centrifugation. Samples were cryopreserved in Dulbecco’s modified Eagle medium (DMEM; Sigma Aldrich, MO) containing 20% dimethyl sulfoxide (Sigma Aldrich, MO) and 20% fetal calf serum (FCS, Sigma Aldrich, MO) and stored at − 80 °C.

**Table 1:** Characteristics of the patient cohorts

	Control	DMF	FTY	GA	NAT	NAT longitudinal
Number of patients	19	21	17	18	20	7
Age (y)	34.2 ± 8.1	37.0 ± 11.9	39.5 ± 9.4	40.1 ± 9.1	35.7 ± 10.3	25.6 ± 6.3
Female sex (%)	73.7	47.6	64.7	50.0	55.0	71.4
EDSS score	2.00 ± 1.40	1.95 ± 1.50	2.76 ± 1.39	1.81 ± 1.43	3.70 ± 2.11	2.64 ± 2.01
MS since (y)	5.07 ± 5.30	5.48 ± 3.89	12.00 ± 4.50	7.50 ± 4.04	9.50 ± 5.45	2.39 ± 2.81
Drug taken since (y)	–	0.73 ± 0.31	1.75 ± 0.99	4.22 ± 2.48	3.57 ± 2.78	–
Previous treatment (*n*)						
Interferon beta	0	5	3	1	10	1
Glatiramer acetate	0	3	2	0	2	1
Natalizumab	0	2	4	0	0	0
Fingolimod	0	0	0	0	2	0
Mitoxantrone	0	0	0	0	0	1
None	19	11	8	17	6	4

Control multiple sclerosis (MS) patients had not been treated with any immunomodulatory drug for at least 6 months when phlebotomy was performed, while dimethyl fumarate (DMF)-, fingolimod (FTY)-, glatiramer acetate (GA)-, and natalizumab (NAT)-treated MS patients were on medication for at least 6 months before sampling. Data are displayed as mean ± standard deviation

*EDSS* Expanded Disability Status Scale, *y* years

### Cell count determination

Immune cell counts from whole blood were determined in our hospital’s routine laboratory. To determine the frequency of the respective immune cell populations (CD4^+^, CD8^+^, and CD19^+^ cells), we first excluded doublets, followed by gating for lymphocytes according to their size and granularity (FSC vs SSC). Thereafter, we gated for living lymphocytes by exclusion of Zombie positive cells, finally determining the frequency of CD4^+^, CD8^+^, and CD19^+^ within all living lymphocytes. We then multiplied the lymphocyte count with the fraction of the lymphocyte subset of interest determined by flow cytometry. Since this approach is based on scatter gating only, there is some room for small inaccuracies which should be considered when evaluating the data.

### PBMC handling and stimulation

For analysis, cells were thawed; washed in DMEM containing 10% FCS, 1% sodium pyruvate (Sigma Aldrich, MO), 1% L-glutamine (Sigma Aldrich, MO), and 0.1% β-mercaptoethanol (Sigma Aldrich, MO); and plated at a concentration of 0.5 × 10^6^ cells/ml in 96-well U-bottom plates (Sarstedt, Germany). For the analysis of activation marker and co-stimulatory molecules, PBMC were stimulated with 2 μg/ml CpG oligodeoxynucleotides (CpG) or 100 pg/ml lipopolysaccharide (LPS) as indicated for 20 h at 37 °C and 5% CO_2_.

To determine the intracellular cytokine content, PBMC were cultured for 12 h in the presence of 1 μg/ml CpG followed by incubation with 500 ng/ml ionomycin, 20 ng/ml phorbol 12-myristate 13-acetate (PMA; both Sigma Aldrich, MO), and the protein transport inhibitor GolgiPlug (BD Bioscience, NJ) for 4 h according to the manufacturer’s recommendations. For the in vitro analysis of NAT-mediated effects, we incubated PBMC of healthy donors for 4 h with various concentrations of NAT or an immunoglobulin G (IgG) 4 isotype control antibody (IGHG4; Biolegend, CA) followed by 40 h simultaneous incubation with NAT/control and 1 μg/ml CpG. Thereafter, GolgiPlug, 500 ng/ml ionomycin, and 20 ng/ml PMA were added for additional 6 h. Geometric mean fluorescent intensity (gMFI) of intracellularly accumulated cytokines was determined via flow cytometry. For the evaluation of apoptosis, PBMC were incubated with 30 μg/ml NAT or isotype control antibody for 72 h.

### Flow cytometry analysis

Prior to antibody incubation, cells were stained with viability dye (Zombie™ dye, 1:500, Biolegend) for live cell/dead cell discrimination and incubated with Fc receptor blocking solution (Human TruStain FcX, BioLegend, CA) to prevent unspecific antibody binding. Extracellular antigens were stained using anti-human cluster of differentiation (CD)4-PE-Cy7, CD8-PE, CD14-PE-CF594 and CD19-FITC/PerCP-Cy5.5, CD20-APC-Cy7, CD25-BV605, CD27-PacificBlue, CD38-FITC, CD80-PE-Cy7, CD150-BV-421, major histocompatibility complex class II (MHC-II)-APC (all Biolegend, CA), CD19-PerCp-Cy5.5, CD40-PE-Dazzle, CD69-FITC, CD86-BV421, and CD95-PE (all BD Biosciences, NJ) antibodies. For analysis of intracellular cytokines, cells were permeabilized by adding fixation/permeabilization solution (Cytofix/Cytoperm, BD Biosciences, NJ) and stained with anti-human interleukin (IL)-6-FITC, IL-10-PE/CF594, and tumor necrosis factor (TNF)-Alexa Flour 700 (all BD Biosciences, NJ) antibodies. Apoptosis was evaluated using propidium iodide-PE and annexin V-FITC (both BioLegend, CA). Samples were analyzed using a LSRII Fortessa; FACS Diva (BD Biosciences) and FlowJo software were used to quantify flow cytometric data.

### B cell proliferation assay

For the analysis of B cell proliferation, B cells were isolated using magnetic-activated cell sorting (MACS; anti-human CD19 MicroBeads, Miltenyi Biotec). After carboxyfluorescein succinimidyl ester (CFSE) staining (BD Biosciences), 60,000 cells/well were plated in 96-well plates and stimulated with anti-human IgG and IgM F(ab)_2_ antibody fragments (20 μg/ml; Jackson Immunoreaearch, PA), anti-human CD40 antibodies (10 μg/ml; BioCell, NH), CpG (0.5 μg/ml), and IL21 (50 ng/ml) for 72 h. Samples were analyzed using a LSRII Fortessa; FACS Diva (BD Biosciences) and FlowJo software were used to quantify flow cytometric data.

### Statistical analysis

For normality testing, we used the D’Agostino & Pearson omnibus normality test; the paired *t* test was used for parametric data, Mann-Whitney *U* tests for non-parametric data, and the Wilcoxon signed-rank tests for longitudinal samples. Statistical significance was defined as *P* < 0.05.

## Results

**Fig. 1:**
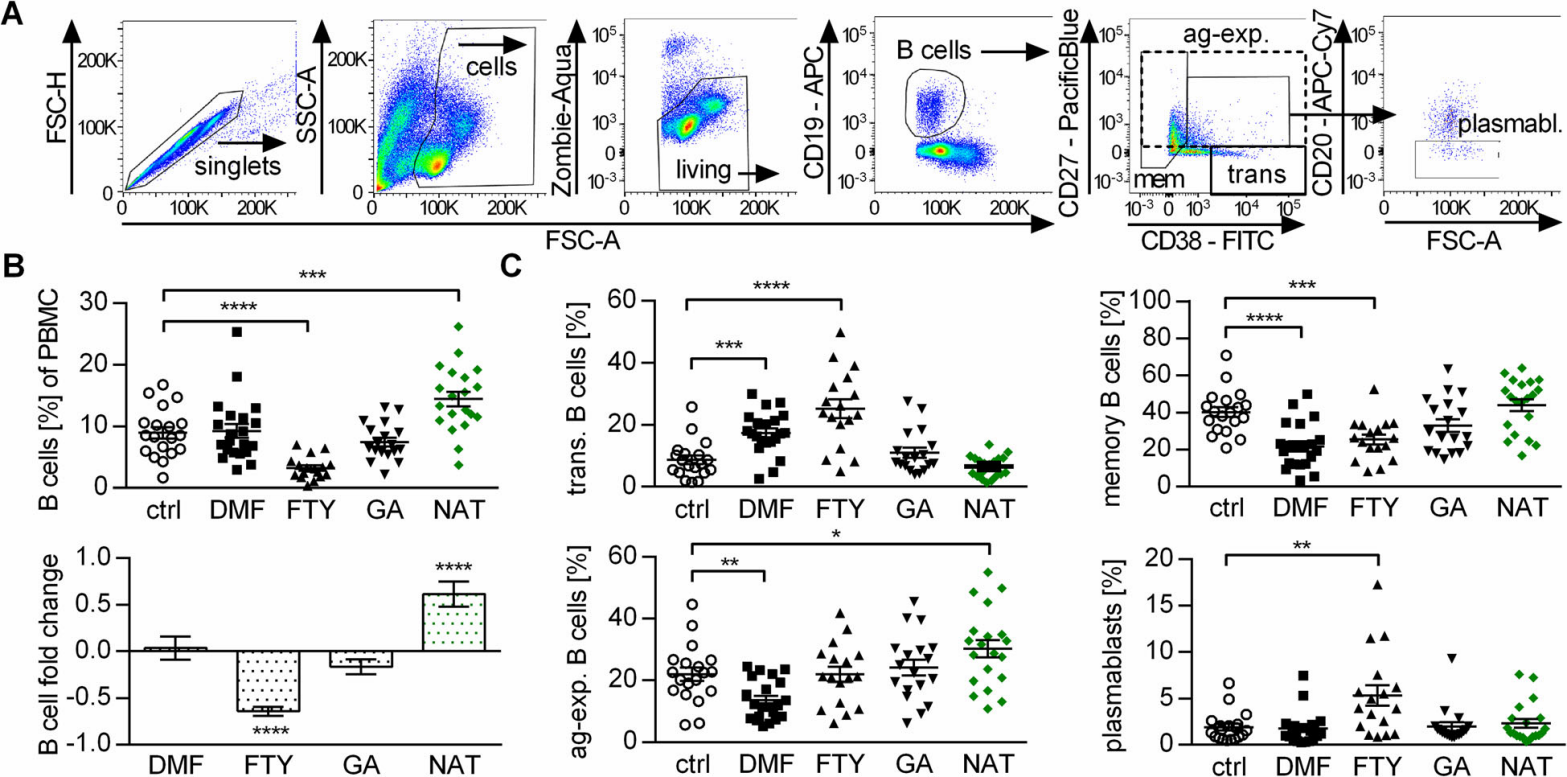
B cell subset frequencies are altered by established MS medications. Peripheral blood mononuclear cells (PBMC) were isolated from controls (*n* = 19; circles) or dimethyl fumarate (DMF; *n* = 21; squares)-, fingolimod (FTY; *n* = 17; triangles up)-, glatiramer acetate (GA; *n* = 18; triangles down)-, and natalizumab (NAT; *n* = 20; diamonds)-treated multiple sclerosis patients. Cells were stained with the respective antibodies and analyzed using flow cytometry. Bars represent standard error of the mean (SEM); * *P* < 0.05; **P < 0.01; ***P < 0.001; ****P < 0.0001; unpaired *t* test. **a** Exemplary gating strategy: within all recorded events, doublets were excluded and living cells were determined using size exclusion and staining with Zombie dye. CD19^+^ B cells were further subdivided into transitional B cells (trans; CD27^−^ CD38^+^), antigen-experienced B cells (ag-exp.; CD27^+^), and memory B cells (mem; CD27^var^; CD38^−^). Within the CD27^+^ CD38^+^ cells, plasmablasts (plasmabl.; CD20^−^ CD27^+^ CD38^+^) were defined as CD20^−^. **b** Mean frequency and fold changes (treated/control 1; e.g., a value of − 0.4 represents a reduction by 40%) ± SEM of CD19^+^ B cells within the PBMC pool, grouped according to the patient’s treatment. **c** Mean frequency ± SEM of transitional B cells, memory B cells, antigen-experienced B cells, and plasmablasts within the B cell pool

First, we investigated the effect of treatment with FTY, NAT, DMF, and GA on the overall abundance of B cells in the blood. Compared to untreated MS patients, the FTY group showed a reduced B cell frequency, NAT treatment resulted in a significant increase of B cells, and both DMF and GA had no detectable effect (Fig. 1a, b). DMF and even more so FTY raised the relative abundance of immature transitional B cells, while the frequency of differentiated memory B cells was correspondingly lower in both groups. DMF treatment was furthermore associated with a reduced frequency of CD27^+^ antigen-experienced B cells, while NAT treatment resulted in a substantial rise of this mature B cell population. Lastly, the proportion of plasmablasts was elevated upon FTY treatment (Fig. 1c). Using this set of parameters, GA treatment exerted no detectable effect on B cells.

**Fig. 2:**
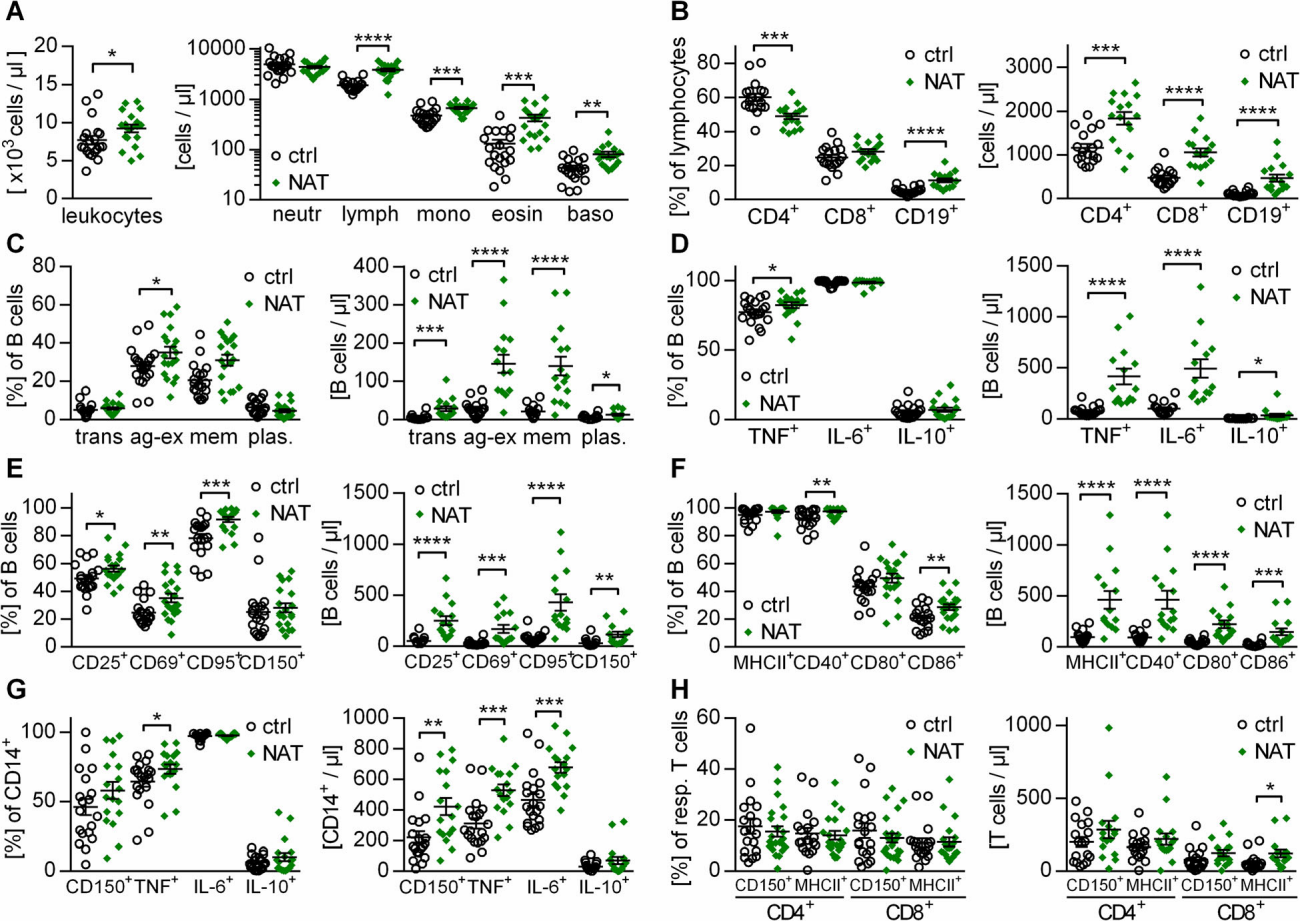
Natalizumab treatment increases the expression of activation markers and TNF production in B cells. Peripheral blood mononuclear cells (PBMC) were isolated from controls (*n* = 19; circles) or natalizumab (NAT; *n* = 20; diamonds)-treated multiple sclerosis patients. Cells were stained with the respective antibodies and analyzed using flow cytometry. Each dot represents the value of an individual patient; bars indicate mean ± standard error of the mean; *P < 0.05; **P < 0.01; ***P < 0.001; ****P < 0.0001; unpaired *t* test for cross-sectional data. **a** Total leukocyte counts and neutrophil, lymphocyte, monocyte, eosinophil, and basophil counts were determined from whole blood. **b**, **c** Cell frequencies and absolute cell counts were determined for **b** CD4^+^ and CD8^+^ T cells and CD19^+^ B cells as well as for **c** B cell subsets such as transitional B cells (trans), antigen-experienced B cells (ag-ex), memory B cells (mem), and plasmablasts (plas.). **d** PBMC were stimulated with 1 μg/ml CpG for 12 h, followed by 4 h of 500 ng/ml ionomycin / 20 ng/ml phorbol 12-myristate 13-acetate (PMA) stimulation. We determined the frequency as well as the absolute number of B cells producing tumor necrosis factor alpha (TNF), interleukin-(IL-)6, and IL-10. **e**, **f** Frequency and absolute number of B cells expressing markers for activation (**e**) and antigen presentation (**f**) were determined after 20 h of 2 μg/ml CpG stimulation. **g** PBMC were pre-stimulated for 12 h with 1 μg/ml CpG, followed by 4 h of 500 ng/ml ionomycin / 20 ng/ml PMA stimulation. We determined the frequency and absolute number of myeloid cells (CD14^+^) producing TNF, IL-6, or IL-10. CD150 expression was determined after 20 h of incubation with 100 pg/ml lipopolysaccharides (LPS). **h** PBMC were incubated with 100 pg/ml LPS for 20 h and determined their expression of CD150 and major histocompatibility complex class II (MHC-II)

**Fig. 3:**
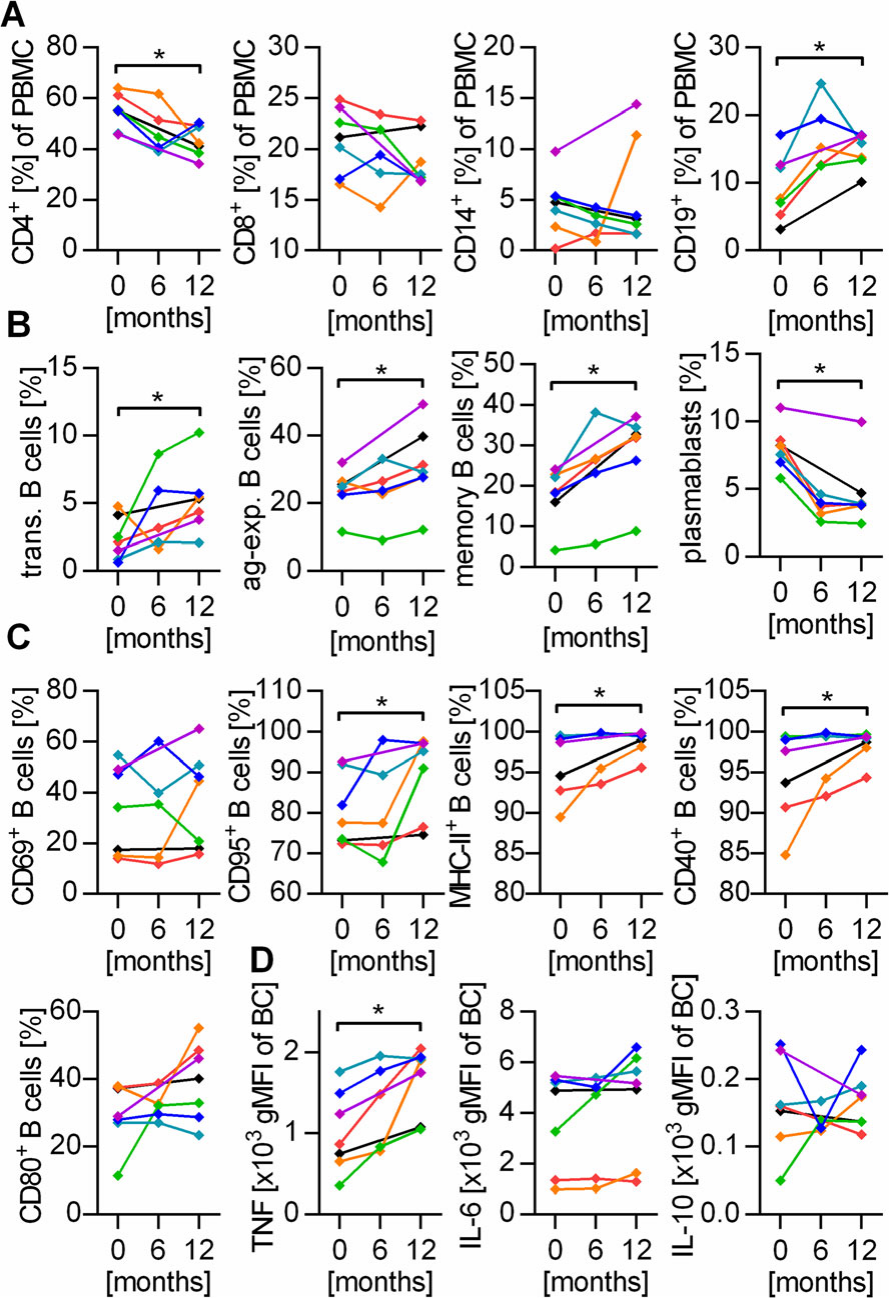
Natalizumab increases pro-inflammatory properties of B cells in longitudinal samples. Blood samples (*n* = 7) were collected before NAT treatment initiation and at the indicated time intervals. Cells were stained with the respective antibodies and analyzed using flow cytometry. Lines connect the values of individual patients. *P < 0.05; Wilcoxon signed-rank test. **a** Frequency of CD4^+^ and CD8^+^ T cells, CD14^+^ phagocytes, and CD19^+^ B cells within all peripheral blood mononuclear cells (PBMC). **b** Frequency of transitional (trans.) B cells, antigen-experienced (ag-exp.) B cells, memory B cells, and plasmablasts within all B cells. **c** The expression of activation markers and molecules involved in antigen presentation was determined after stimulation with 2 μg/ml CpG for 20 h. **d** PBMC were pre-stimulated for 12 h with 1 μg/ml CpG, followed by 4 h of 500 ng/ml ionomycin / 20 ng/ml phorbol 12-myristate 13-acetate stimulation. Cytokine production of B cells (BC) was quantified using the geometric mean fluorescent intensity (gMFI) of the respective cytokine

Analyzing the effect of NAT in detail, we found that NAT treatment was associated with an incline of the total number of leukocytes (Fig. 2a left), including lymphocytes, monocytes, eosinophils, and basophils (Fig. 2a right). Within the enriched fraction of lymphocytes, the frequency of B cells was proportionally increased, while CD4^+^ T cells were compensatory reduced (Fig. 2b left). However, when calculating absolute numbers, all investigated immune cell populations were significantly elevated upon NAT treatment (Fig. 2b right). Furthermore, B cells from NAT-treated patients were distinctly enriched in antigen-activated and memory B cells (Fig. 2c), showed a higher production of pro-inflammatory cytokines (Fig. 2d), and presented with an elevated frequency and total number of activated CD25^+^, CD69^+^, and CD95^+^ cells (Fig. 2e). While the expression of MHC-II remained unchanged, the level of the co-stimulatory molecules CD40, CD80, and CD86 was increased on B cells upon NAT treatment (Fig. 2f). In conjunction, this finding revealed a rise in all investigated immune cell subpopulations and pointed towards a predominant increase in pro-inflammatory B cell subsets upon NAT treatment. In line with this data, the longitudinal analysis of interrelated samples collected prior and after NAT treatment initiation confirmed our observations regarding memory, antigen-experienced (CD27^+^), CD40^+^, CD95^+^, and TNF^+^ B cells (Fig. 3) and consolidated the assumption that NAT treatment triggers the activation and pro-inflammatory differentiation of B cells. In addition, NAT treatment was associated with an increase in the production of pro-inflammatory TNF and IL-6 by CD14^+^ myeloid cells (Fig. 2g) as well as an enhanced expression of the activation marker CD150, an effect which was even more pronounced when absolute cell numbers were analyzed. Only minor effects could be detected regarding the assessed parameters on T cells, where the number of MHC-II^+^ CD8^+^ T cells was slightly elevated in NAT-treated patients (Fig. 2h).

**Fig. 4:**
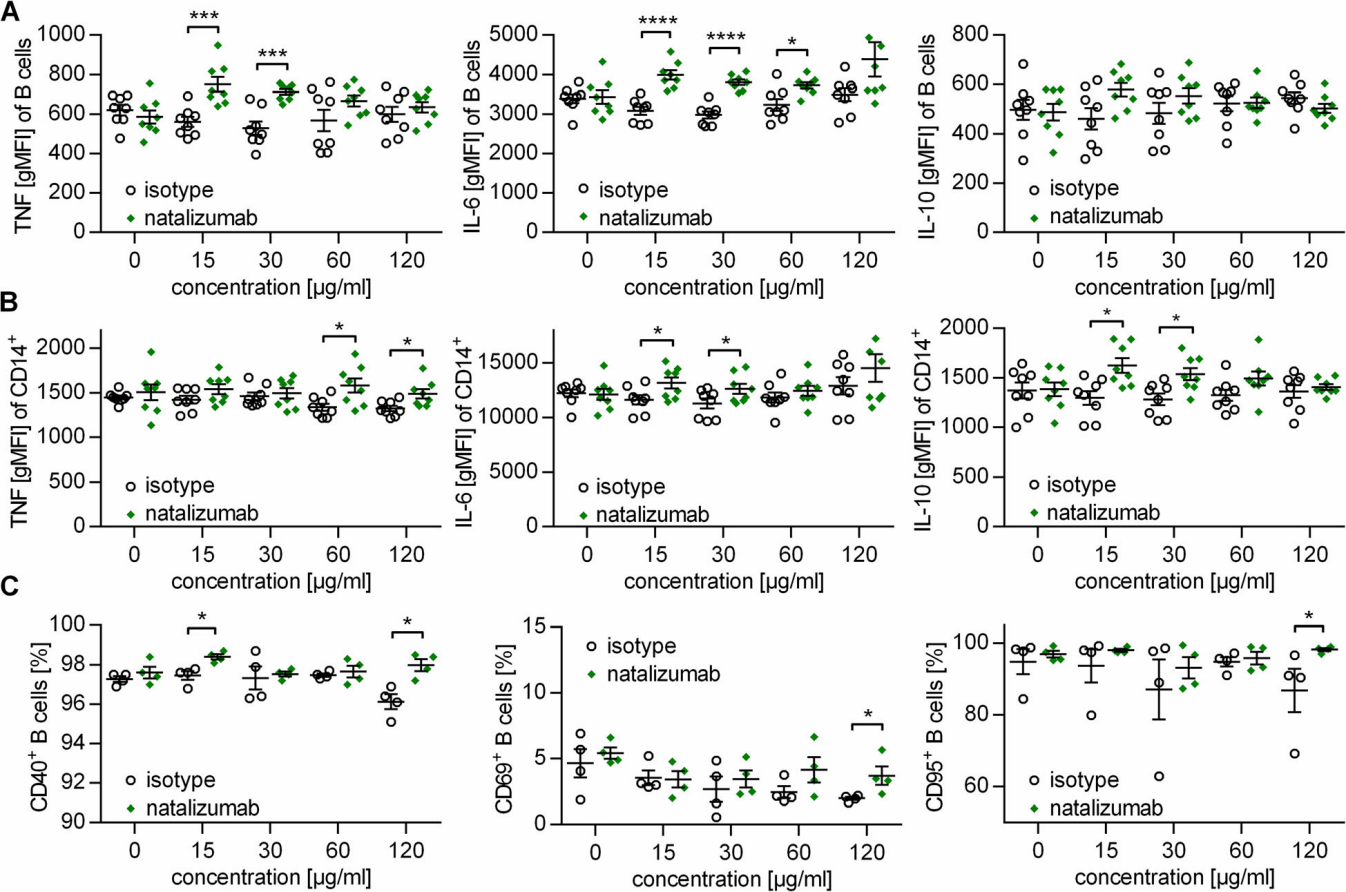
Natalizumab increases TNF and IL-6 production of B cells in vitro. Peripheral blood mononuclear cells (PBMC) were collected from healthy donors (*n* = 8). Two hundred thousand PBMC were pre-incubated for 4 h with increasing concentrations of natalizumab (NAT) or an isotype control IgG4 antibody followed by 40 h simultaneous incubation with NAT/control and 1 μg/ml CpG. Each dot represents the values of one healthy donor. Whiskers indicate mean ± standard error of the mean; *P < 0.05; ***P < 0.001; ****P < 0.0001; unpaired *t* test. **a**, **b** Thereafter, GolgiPlug, 500 ng/ml ionomycin, and 20 ng/ml phorbol 12-myristate 13-acetate (PMA) were added for an additional 6 h. Geometric mean fluorescent intensity (gMFI) of intracellularly accumulated cytokines (tumor necrosis factor (TNF), interleukin (IL)-6 and IL-10) in **a** B cells (CD19^+^) and **b** CD14^+^ myeloid cells was determined via flow cytometry. **c** Without further stimulation, expression of activation markers and co-stimulatory molecules on B cells were determined using flow cytometry

**Fig. 5:**
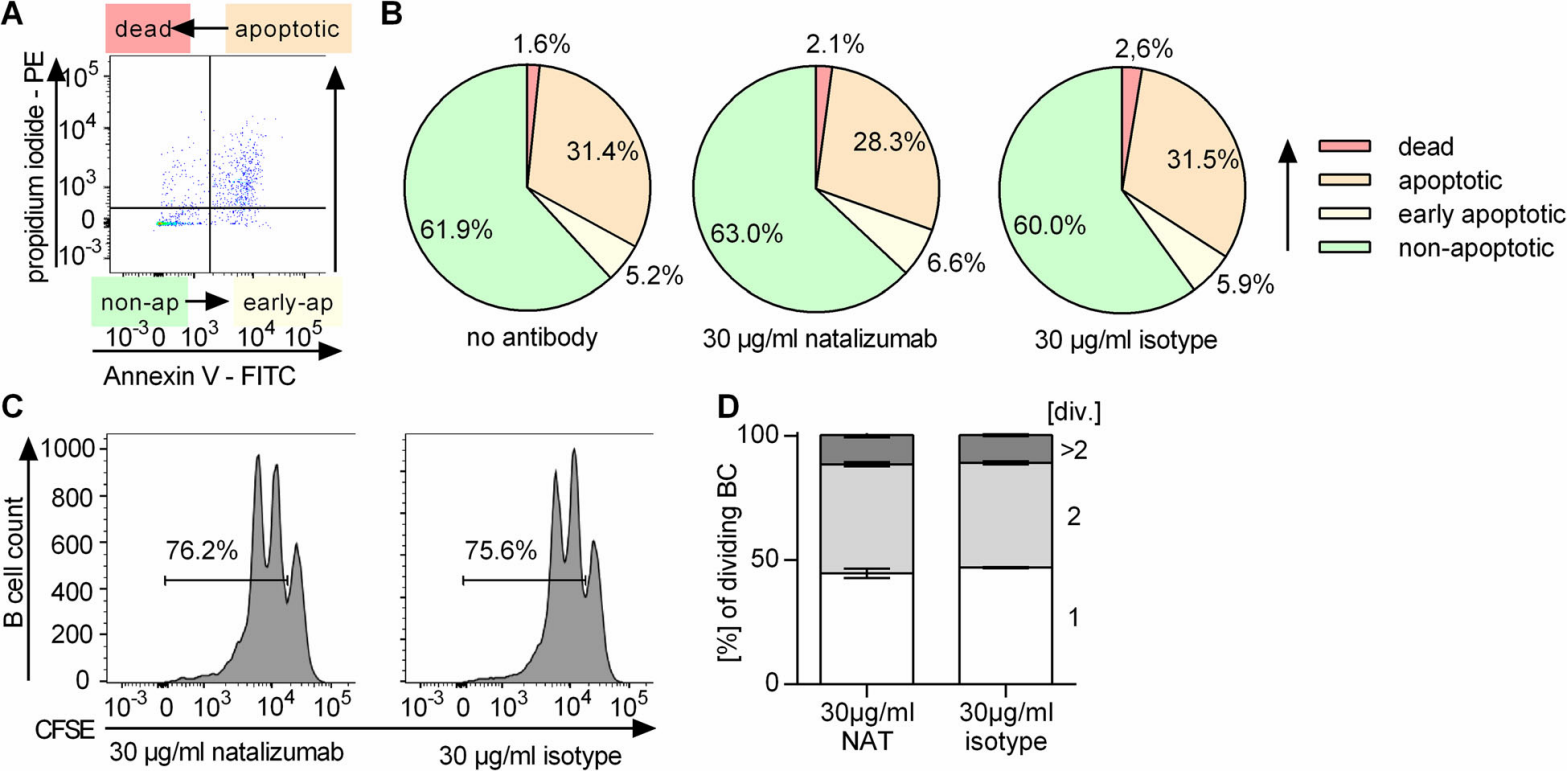
Neither apoptosis nor proliferation of B cells is affected by in vitro natalizumab exposure. Peripheral blood mononuclear cells (PBMC) were collected from healthy donors (*n* = 6). **a**, **b** PBMC were incubated in vitro for 72 h with no antibody, 30 μg/ml natalizumab (NAT), or an IgG4 isotype control antibody. To evaluate apoptosis of CD19^+^ B cells, cells were stained with anti-CD19 antibody, propidium iodide (PI), and annexin V (AV). Non-apoptotic B cells (PI^−^ AV^−^), early apoptotic B cells (PI^−^ AV^+^), apoptotic B cells (PI^+^ AV^+^), and dead B cells (PI^+^ AV^−^) were distinguished. **a** Exemplary dot plot and **b** respective pie charts showing the mean frequency of the analyzed parameters. **c**, **d** B cells were isolated from PBMC using magnetic-activated cell sorting (MACS). After carboxyfluorescein succinimidyl ester (CFSE) staining, they were stimulated with anti-human IgG and IgM F(ab)_2_ fragments (20 μg/ml), anti-human CD40 (10 μg/ml), CpG (0.5 μg/ml), and interleukin (IL)-21 (50 ng/ml) for 72 h in the presence of 30 μg/ml of natalizumab or IgG4 isotype control antibody. Frequency of dividing cells was determined by CFSE dilution using flow cytometry. **c** Representative histograms (**d**) and frequency of B cells showing 1, 2, or > 2 divisions (div.); bars indicate mean ± standard error of the mean

In order to study the immune-stimulating effect of NAT mechanistically, we cultured whole PBMC of healthy donors with increasing concentrations of NAT (0–120 μg/ml) in vitro, which were chosen according to reported serum levels in treated patients [4]. In line with the patient’s ex vivo data, in vitro NAT exposure enhanced the production of pro-inflammatory TNF and IL-6 by B cells and CD14^+^ myeloid cells (Fig. 4a, b) and upregulated the expression of CD40, CD69, and CD95 on B cells (Fig. 4c). To explain mechanistically how the aforementioned enrichment of B cells in NAT-treated patients may occur, we investigated the proliferation and apoptosis behavior of these cells upon NAT treatment. Within the chosen culture period, NAT exposure to PBMC or purified B cells exerted an effect on neither B cell frequency (no antibody 11.1 ± 0.7%, NAT 12.0 ± 0.9%; isotype 11.8 ± 1.1%), nor their apoptosis rate (Fig. 5a, b) or proliferation (Fig. 5c-d).

## Discussion

In this manuscript, we aimed to investigate in a parallel approach how commonly used MS medications affect B cells, a procedure allowing us to compare treatment effects directly without inter-study discrepancies. Despite the fact that all investigated agents have proven efficacy in large clinical trials, our data showed that DMF, FTY, GA, and NAT exerted differential, in parts even opposing effects on the composition and properties of peripheral B cells. Confirming previous studies, our data showed that GA treatment has no detectable effect on B cell maturation and differentiation [5], while both DMF [6] and FTY [7] suppressed the prevalence and function of mature B cells and increased the relative frequency of their immature phenotypes.

NAT treatment on the contrary was associated with a substantial expansion of all leukocytes in blood, but most strikingly of mature B cell subsets. In an attempt to investigate whether this enrichment may ascribe to changes in cell survival, we challenged B cells in vitro with NAT, but detected neither effects on apoptosis nor proliferation. Various other explanations have been proposed to explain the rise of B cells in blood. Based on the observation that particularly the number of CXCR3^+^ B cells is increased in this compartment [8], Saraste et al. suggested that NAT treatment detains B cells with a high migratory capacity in the blood stream, which would—without treatment—extravasate into the inflamed tissue. Others though claimed that NAT treatment perturbs the homing of mature B cell subsets into secondary lymphoid organs, which increases their prevalence in the circulation [9]. Our in vitro observation that NAT exposure enhances neither apoptosis nor proliferation of B cells may add an additional explanation why B cells are enriched upon NAT treatment.

Besides these assumed effects on cellular compartmentalization, we observed that in vivo and in vitro treatment with NAT resulted in B cells with an elevated production of pro-, but not anti-inflammatory cytokines and enhanced expression of activation marker and co-stimulatory molecules. Especially the latter setting indicated that NAT exerted a direct stimulating effect on B cells, possibly mediated by the bidirectional signaling effect upon binding of the NAT to CD49d [10, 11], a mode of action suggested to increase IL-2, interferon-γ, and IL-17 production of purified CD4^+^ T cells in vitro [12]. In this context, it was shown that NAT therapy modulates microRNA and pro-inflammatory cytokine expression in T cells of MS patients [13, 14], suggesting underlying epigenetic processes. In comparison with the described stimulating impact of NAT on T cells, we observed a much stronger effect on B cells in our study. This may be explained by the higher expression of CD49d on B cells than T cells [15]. Furthermore, CD49d is as well expressed on CD14^+^ myeloid cells [16], a fact possibly explaining why the production of pro-inflammatory cytokine by these cells is also enhanced upon NAT treatment.

We assume that the here described increase in B cell number and activation upon NAT treatment is not just an epiphenomenon but has clinical implications. This assumption is fueled by the observation that high B cell frequencies after NAT treatment are associated with ongoing disease activity [17]. Along the same lines, a recent report showed that B cells are enriched in the CNS parenchyma of patients experiencing relapses after NAT therapy cessation [18] placing focus on the question whether a prompt therapeutic follow-up with B cell suppressive agents can decrease the risk of such relapses. Indeed, first investigations showed that patients receiving the B cell-inhibiting agent FTY as follow-up treatment after NAT experience less frequent relapses than patients receiving GA [19] or no ensuing treatment [20]. B cell depletion with rituximab was even more effective than FTY as a post-NAT treatment [21], underscoring the presumed key role of B cells in relapses after NAT cessation.

Moreover, the development of extra nodal large B cell lymphomas that has been described in some NAT-treated patients [22] may be linked to our observations regarding B cell activation and proliferation.

Lastly, the expansion of peripheral B cells with presumed pathogenic properties plausibly explains why NAT worsens neuromyelitis optica [23, 24]. In this regard, it needs to be evaluated how patients with other CNS demyelinating diseases, which show a prominent B cell contribution, such as myelin oligodendrocyte glycoprotein antibody-associated encephalomyelitis [25], or MS patients solely and consistently responding to plasmapheresis as relapse intervention [26] respond to NAT.

### Limitations

We are aware that the small cohort size is limiting this study and that future investigations, which will include a larger population, extended clinical readouts, and a longer follow-up, should substantiate our data. More detailed in vitro experiments on isolated B cells may clarify if the observed pro-inflammatory effects are induced directly on B cells or indirectly via other immune cells.

## Conclusions

Established MS therapeutics exert fundamentally opposing effects on B cells, reaching from their inhibition (DMF, FTY) to substantial activation (NAT). Possible clinical consequences of these complex alterations yet need to be established.

## Data Availability

The data that support the findings of this study are available from the corresponding author upon reasonable request.

## Abbreviations

CD: Cluster of differentiation
CFSE: Carboxyfluorescein succinimidyl ester
CNS: Central nervous system
CpG: CpG oligodeoxynucleotides
DME: Dulbecco’s modified Eagle medium
DMF: Dimethyl fumarate
EDSS: Expanded disability status scale
FCS: Fetal calf serum
FTY: Fingolimod
GA: Glatiramer acetate
gMFI: Geometric mean fluorescent intensity
HLA-DR: Human leukocyte antigen – DR isotype
IgG: Immunoglobulin G
IL: Interleukin
LPS: Lipopolysaccharides
MACS: Magnetic-activated cell sorting
MHC-II: Major histocompatibility complex class 2
MS: Multiple sclerosis
NAT: Natalizumab
PBMC: Peripheral blood mononuclear cells
PMA: Phorbol 12-myristate 13-acetate
TNF: Tumor necrosis factor
